# A novel mitochondrial genome of *Arborophila* and new insight into *Arborophila* evolutionary history

**DOI:** 10.1371/journal.pone.0181649

**Published:** 2017-07-25

**Authors:** Chaochao Yan, Biqin Mou, Yang Meng, Feiyun Tu, Zhenxin Fan, Megan Price, Bisong Yue, Xiuyue Zhang

**Affiliations:** 1 Key Laboratory of Bio-Resources and Eco-Environment (Ministry of Education), College of Life Sciences, Sichuan University, Chengdu, P.R. China; 2 Institute of Wildlife Conservation, Jiangxi Academy of Forestry, Nanchang, P.R. China; 3 Sichuan Key Laboratory of Conservation Biology on Endangered Wildlife, College of Life Sciences, Sichuan University, Chengdu, P.R. China; National Cheng Kung University, TAIWAN

## Abstract

The lineage of the Bar-backed Partridge (*Arborophila brunneopectus*) was investigated to determine the phylogenetic relationships within *Arborophila* as the species is centrally distributed within an area covered by the distributions of 22 South-east Asian hill partridge species. The complete mitochondrial genome (mitogenome) of *A*. *brunneopectus* was determined and compared with four other hill partridge species mitogenomes. NADH subunit genes are radical in hill partridge mitogenomes and contain the most potential positive selective sites around where variable sites are abundant. Together with 44 other mitogenomes of closely related species, we reconstructed highly resolved phylogenetic trees using maximum likelihood (ML) and Bayesian inference (BI) analyses and calculated the divergence and dispersal history of *Arborophila* using combined datasets composed of their 13-protein coding sequences. *Arborophila* is reportedly be the oldest group in Phasianidae whose ancestors probably originated in Asia. *A*. *rufipectus* shares a closer relationship with *A*. *ardens* and *A*. *brunneopectus* compared to *A*. *gingica* and *A*. *rufogularis*, and such relationships were supported and profiled by NADH dehydrogenase subunit 5 (*ND5*). The intragenus divergence of all five *Arborophila* species occurred in the Miocene (16.84~5.69 Mya) when there were periods of climate cooling. We propose that these cooling events in the Miocene forced hill partridges from higher to lower altitudes, which led to geographic isolation and speciation. We demonstrated that the apparently deleterious +1 frameshift mutation in NADH dehydrogenase subunit 3 (*ND3*) found in all *Arborophila* is an ancient trait that has been eliminated in some younger lineages, such as Passeriformes. It is unclear of the biological advantages of this elimination for the relevant taxa and this requires further investigation.

## Introduction

Species of the *Arborophila* (commonly: hill partridge) are found in Asia from the Himalayas eastwards to Taiwan and south to Java. *Arborophila* is a very diverse genus, being the second most species rich genus within Galliformes [1–3]. Hill partridges are fairly small and their body typically ranges between 24 and 30 cm, while their beaks and tails are of equal length [2]. These small partridges are often brightly marked, but this marking provides camouflage in the leaf litter of eastern and southern Asia forests. Hill partridges are plump-bodied with thick necks and moderately long legs, stronger and longer tarsometatarsal, and rounded and rather short wings. Thus hill partridges are poor fliers with low aerial maneuverability.

The *Arborophila* genus contains over 20 species with 10 of those being found in China [2,4]. In 1998, eight of the 10 Chinese hill partridge species were listed in the *China red data book of endangered animals* as ‘Rare’ or ‘Endangered’ [5,6], among which the Sichuan Hill Partridge (*A*. *rufipectus*) and Hainan Partridge (*A*. *ardens*) are currently ranked as Endangered and Vulnerable, respectively, by IUCN Red List of Threatened Species [6]. Although some of the hill partridge species have a relatively wide distribution and inhabit a variety of habitats, their poor flying ability and ground-dwelling nature makes them vulnerable to human activities, non-indigenous predators and rapid loss of habitat and are thus rare within their respective ranges.

Research regarding the taxonomic status and interspecific relationships of *Arborophila*, is still in its infancy and is currently being debated [7–9]. Recent studies tend to place the *Arborophila* near the root position of Phasianidae [3,10–12], however, the phylogenetic relationship within *Arborophila* has been little studied.

Mitochondrial DNA (mtDNA) has frequently and widely been used as an ideal marker to resolve intractable phylogenies, because it typically accumulates mutations at a faster rate than nuclear DNA [8]. The accumulation of mutations makes it more effective to resolve relationships among recently diverged species or populations. However this rapid accumulation of mutations makes mtDNA incompatible to resolve older relationships because the more variable DNA sequences from remote species can easily become saturation [13]. Therefore we applied careful testing to avoid saturation occurring in our dataset. Another limitation of mtDNA is that it is considered prone to introgression among species and only reflects a one-sided perspective on the ancestry [14]. However, mtDNA has merits such as lack of recombination and is easily accessible. Moreover, the high rates of anatomical evolution match the rapid mitoDNA evolution in birds somehow[4,15–17].

**Fig 1:**
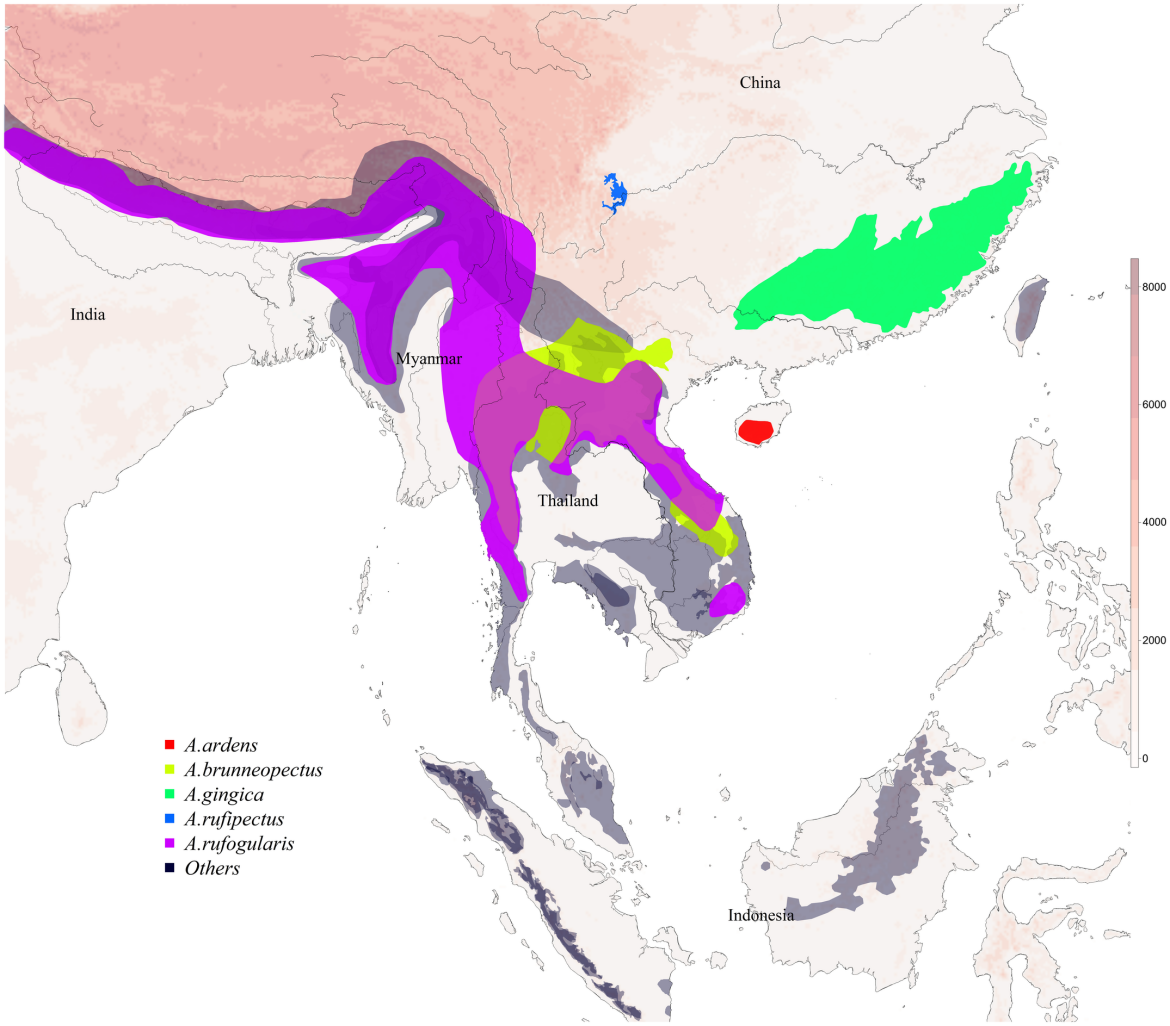
Geographic distribution of the five hill partridges within this study in comparison to the overall distribution of the other 17 hill partridges in South-east Asia. The five studied hill partridge distributions have been assigned different colors while the others 17 are all colored midnight blue. Elevation information (altitude: meters above sea level) is represented by different heat colors.

Unlike the phylogenic distribution of *Arborophila*, the geographic distribution is relatively well understood. The widely distributed Bar-backed Partridge (*A*. *brunneopectus*) ranges across an area that sits centrally to the distributions of 22 extant hill partridge species, and overlaps with three of the 22 species (Fig 1). It is reported that *A*. *brunneopectus* shares a closer relationship with *A*. *ardens*, although the number of intermediate species between *A*. *brunneopectus* and *A*. *ardens* is unknown. Complete ecological data of these species are lacking [18–20].

To date, only four mitogenomes within the genus *Arborophila* have been determined, while two are incomplete and lackspecific descriptions [21]. In this study, we determined the complete mitochondrial genome of *A*. *brunneopectus*. We also reconstructed the phylogenetic tree of 45 related birds and estimated contributions of every single mitogene to the consensus tree. Based on the divergence time and biogeographic data of the studied species, a hypothesis model of settlement and migration of hill partridge species was proposed. We also examined and discussed the relationship of variable sites and positive selective sites as well as the +1 frameshift mutation in NADH dehydrogenase subunit 3 (*ND3*) through the mitochondrial DNA alignments. The publication of the mitogenome of *A*. *brunneopectus* will greatly contribute to understanding of relationships within *Arborophila*.

## Materials and methods

### DNA extraction, amplification and sequencing

The protocol of this research has been reviewed and approved by the Ethics and Experimental Animal Committee of Sichuan Institute of Zoology, Chinese Academy of Science. The Bar-backed Partridge sample was offered by Sichuan Key Laboratory of Conservation Biology on Endangered Wildlife. Total genomic DNA was extracted from breast muscle tissue using standard phenol/chloroform methods [22]. Target segments were amplified with a long and accurate polymerase chain reaction (LA-PCR) technique according to the manufacturer's instructions (TaKaRa, China). For gaps or low quality terminus between target segments, normal PCR was applied as supplementary to complete the sequencing. Twenty pairs of primers were used to amplify our mtDNA fragments, among which the first eight pairs of primers were used in LA-PCR while the remaining 12 pairs were used in normal PCR. The primers used for both PCR amplification and sequencing are listed in S1 Table. LA-PCR reaction was carried out in a 25μl reaction volume containing 100ng template DNA, 1×LA PCR buffer (TaKaRa), 0.2mM dNTP, 2.5mM MgCl_2_, 1μM each primer, 1.0U LA Taq polymerase (TaKaRa). After a pre-denaturation at 95°C for 4min, 35 thermal cycle were carried out with denaturation at 94°C for 30s, annealing at optimal temperature based on each pair of primers for 45s, and extension at 72°C for 1-2min, and after the thermal cycle, a final extension was executed at 72°C for 10min. LA-PCR products were electrophoresed on a 1.0% Agarose Gel and purified with the DNA Agarose Gel Extraction Kit (Omega, USA). The purified fragments were directly sequenced from both strands using primer walking method on the ABI 373/377 capillary sequencer. To fill gaps between fragments, normal PCR was also carried out with corresponding PCR buffer, Taq polymerase in PCR master mix and relatively short extension time according to lengths of their target segments in thermal cycle.

The electropherogram had no double peaks throughout the whole mitogenome and the sequence fragments of genes showed no frameshift mutation stop codons when translated into amino acid except *ND3*. In addition, the total cellular DNA was extracted from muscle tissue where it is particularly rich in mtDNA. For those reasons, the data represents the true mtDNA sequence rather than nuclear mitochondrial pseudogenes.

### Sequences analyses

Original sequences were assembled in Lasergene SeqMan Pro (version7.1.0 DNASTAR, Inc., Madison, WI, USA) and optimized manually. To annotate the mitochondrial genome, tRNAscan-SE 1.21 was used to determine positions of 20 tRNAs in organellar (mitochondrial/chloroplast) tRNAs search mode [23]. Remarkably, the tRNA-Trp was annotated as tRNA-SeC (Selenocysteine tRNAs) in this program and the other tRNA-Ser (GCT) was undetected but found through homological alignment and determined by checking its second structure. The locations of protein-coding genes and ribosomal RNA (rRNA) genes were determined by comparison with known homological sequences.

As a typical character of *Arborophila* mitogenomes, the widely emerged extra 174^th^ cytosine insertion in gene *ND3* was carefully investigated in our five mitogenomes, then extended to all birds accessible in Genbank (S3 Table). Subsequently, these sequences were aligned in MEGA5 followed by removing redundant sequences. The distribution of the two types of birds categorized by *ND3* with extra cytosine (WEC) or without extra cytosine (WOC) was determined and further divided according to their taxonomic orders. The relationships of these orders were drawn mainly from references to recent large scale molecular basedstudies [24–26]. It should be noted that, not all sequence authors are aware of the existence of a +1 frameshift mutation in the avian mitogene and may simply remove the insertion, so that some species exhibiting WOC *ND3* may have misled our results [7,27].

### Phylogenetic and biogeographic analyses

**Fig 2:**
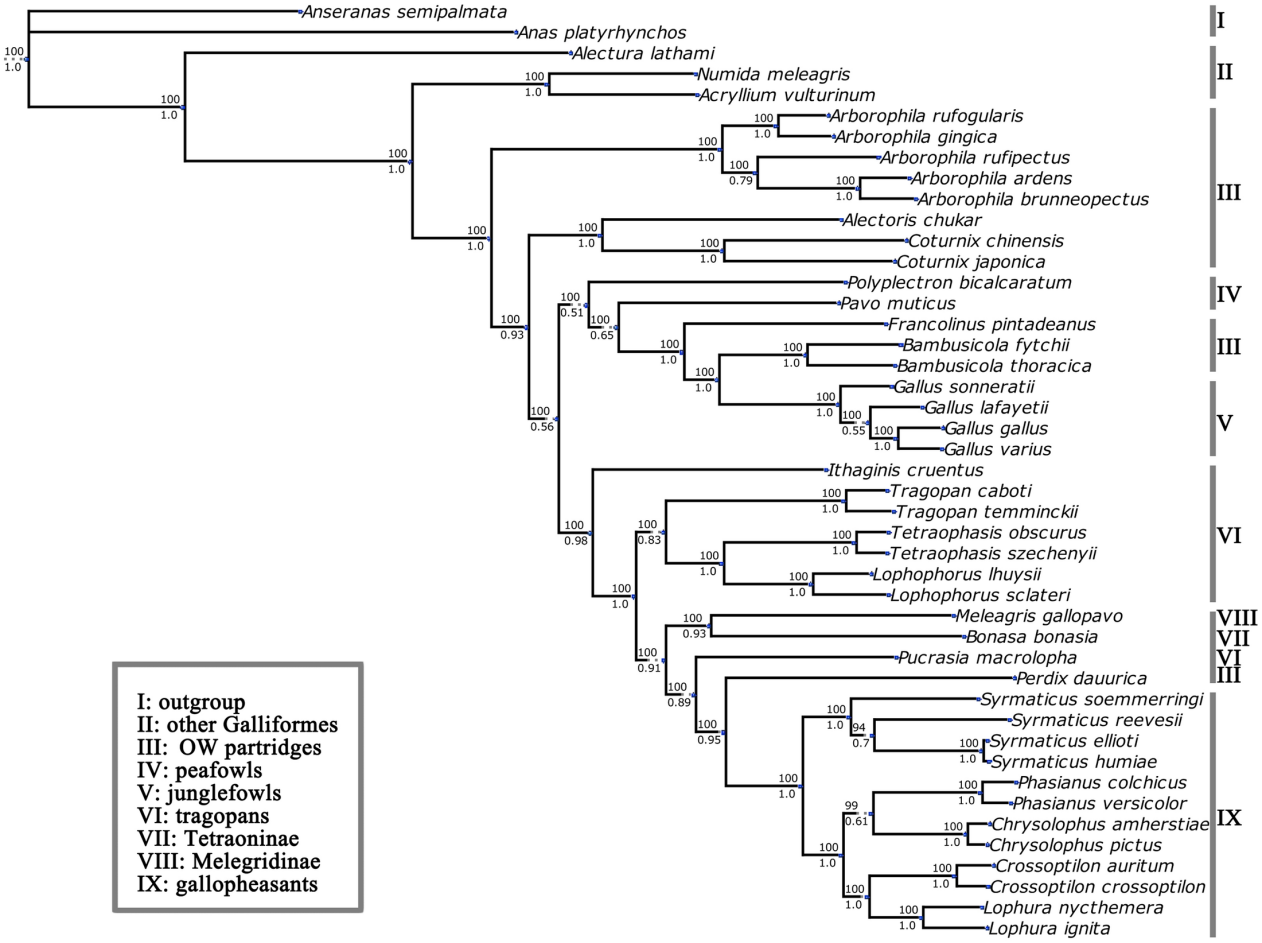
Molecular phylogenetic tree based on 13 mitochondrial protein-coding genes using Bayesian inference (BI) and maximum likelihood (ML) analysis. The numbers beside the nodes are Bayesian posterior probabilities and ML bootstrap proportions. *Anseranas semipalmata* and *Anas platyrhynchos* were set as outgroups.

Our sequenced genome was applied as a query using nucleotide BLAST online to produce a robust phylogenetic tree. We preferentially chose the unique sequence curated by NCBI staff from the nearly 150 homological results and finally got those 44 showed in Table 1. In these species, all the five main lineages (OW partridges, peafowls, junglefowls, tragopans and gallopheasants) of Phasianidae have representative species (Fig 2). The traditional non-pheasant species *Alectura lathami* and *Meleagris gallopavo* were also included in our alignment. However, some genus *Argusianus* were missing and *Polyplectron* were sparse because of there are no complete mitogenomes to date. *Anseranas semipalmata* and *Anas platyrhynchos* were set as outgroups as they were neither too far nor too close to the ingroup in evolution. Moreover, they were proved powerful in constructed robust ingroup relationships in our study. To confirm whether trees inferred from mitoDNA differed from nuclear DNA trees, we collected 35 of the 45 species who shared six nuclear intron sequences amplified in Wang’s study [28].

**Table 1:** Taxa and sequence Genbank accession numbers of the 45 species used for phylogenetic analysis.

Family/subfamily	Species and common name	Genbank accession NO.	Tranditional taxonomy
Anatidae	*Anas platyrhynchos*	NC_009684	Outgroup
Megapodiidae	*Alectura lathami*	NC_007227	Megapodiidae
Meleagridinae	*Meleagris gallopavo*	NC_010195	Meleagrididae
Perdicinae	*Alectoris chukar*	FJ752426	Old World quails
Perdicinae	*Arborophila gingica*	FJ752425	Old World quails
Perdicinae	*Arborophila rufipectus*	NC_012453	Old World quails
Perdicinae	*Arborophila rufogularis*	FJ752424	Old World quails
Perdicinae	*Arborophila ardens*	NC_022683.1	Old World quails
Perdicinae	*Arborophila brunneopectus*	NC_022684.1*	Old World quails
Perdicinae	*Bambusicola fytchii*	FJ752423	Old World quails
Perdicinae	*Bambusicola thoracica*	NC_011816	Old World quails
Perdicinae	*Coturnix chinensis*	NC_004575	Old World quails
Perdicinae	*Coturnix japonica*	NC_003408	Old World quails
Perdicinae	*Francolinus pintadeanus*	NC_011817	Old World quails
Perdicinae	*Perdix dauurica*	FJ752431	Old World quails
Perdicinae	*Tetraophasis obscurus*	NC_018034	Tragopans
Perdicinae	*Tetraophasis szechenyii*	FJ799728	Tragopans
Phasianinae	*Chrysolophus amherstiae*	FJ752434	Gallopheasants
Phasianinae	*Chrysolophus pictus*	NC_014576	Gallopheasants
Phasianinae	*Crossoptilon auritum*	NC_015897	Gallopheasants
Phasianinae	*Crossoptilon crossoptilon*	NC_016679	Gallopheasants
Phasianinae	*Gallus gallus*	NC_001323	Junglefowls
Phasianinae	*Gallus lafayetii*	NC_007239	Junglefowls
Phasianinae	*Gallus sonneratii*	NC_007240	Junglefowls
Phasianinae	*Gallus varius*	NC_007238	Junglefowls
Phasianinae	*Ithaginis cruentus*	NC_018033	Junglefowls
Phasianinae	*Lophophorus lhuysii*	NC_013979	Tragopans
Phasianinae	*Lophophorus sclateri*	FJ752432	Tragopans
Phasianinae	*Lophura ignita*	NC_010781	Gallopheasants
Phasianinae	*Lophura nycthemera*	NC_012895	Gallopheasants
Phasianinae	*Phasianus colchicus*	NC_015526	Gallopheasants
Phasianinae	*Phasianus versicolor*	NC_010778	Gallopheasants
Phasianinae	*Pavo muticus*	NC_012897	Peafowls
Phasianinae	*Polyplectron bicalcaratum*	NC_012900	Peafowls
Phasianinae	*Pucrasia macrolopha*	FJ752429	Tragopans
Phasianinae	*Syrmaticus ellioti*	NC_010771	Gallopheasants
Phasianinae	*Syrmaticus humiae*	NC_010774	Gallopheasants
Phasianinae	*Syrmaticus reevesii*	NC_010770	Gallopheasants
Phasianinae	*Syrmaticus soemmerringi*	NC_010767	Gallopheasants
Phasianinae	*Tragopan caboti*	NC_013619	Tragopans
Phasianinae	*Tragopan temminckii*	FJ752427	Tragopans
Tetraoninae	*Bonasa bonasia*	FJ752435	Tetraoninae
Numididae	*Acryllium vulturinum*	NC_014180	Numididae
Anseranatidae	*Anseranas semipalmata*	NC_005933	outgroup
Numididae	*Numida meleagris*	NC_006382	Numididae

*the sequence determined in this study

After eliminating terminators, the 13 protein-coding genes sequences of each species were concatenated using custom made Perl scripts and were aligned in MAGE 5.2 [29]. The NADH dehydrogenase subunit 6 (*ND6*) gene was included. Although it is encoded by the light-strand that has a significantly different base composition compared to heavy-chain [30], it still fits the best substitution model of all and can develop Bayesian posterior probabilities (BBP) and bootstrap proportions (BSP) in both Bayesian inference (BI) and maximum likelihood (ML) phylogenetic trees. The alignment was checked by comparing with several sequences aligned and concatenated manually. All sites with alignment gaps or missing/ambiguous data were excluded making all sequences a uniform 11313bp.

In order to investigate the possibility of a bias owing to substitution saturation, we estimated the substitution saturation of alignments with Xia’s [31] method and plotted transitions and transversions against pairwise sequence divergence using 13 protein-coding mito-genes with DAMBE 5.3.10 [32]. Codon positions 1 and 2 were not saturated. Codon position 3 was saturated only when adding outgroups. The third codon position was not as constrained as the other two positions according to the Wobble hypothesis when tRNA base pairing. Furthermore, the results illustrated that our alignments had little substitute saturate (ISS > ISS.C, p<0.001) (S1 Fig), and are suitable for phylogenetic analyses.

jModelTest 2.1.1 [33] was used in our combined datasets to select the preferred models of evolution for Bayesian inference (BI) under the Akaike Information Criterion [34,35]. The GTR+I+G model was chosen as the best model for the concatenated datasets. Two partitioned schemes, three codon position to be separated or 13 protein-coding genes to be divided into 3 groups: 1) ATP synthase F0 subunit 6 (*ATP6*), ATP synthase F0 subunit 8 (*ATP8*), cytochrome b (*CYTB*), NADH dehydrogenase subunit 1 (*ND1*), NADH dehydrogenase subunit 2 (*ND2*), *ND3*, NADH dehydrogenase subunit 4 (*ND4*), NADH dehydrogenase subunit 4L (*ND4L*), NADH dehydrogenase subunit 5 (*ND5*); 2) cytochrome c oxidase subunit 1 (*COX1*), cytochrome c oxidase subunit 2 (*COX2*), cytochrome c oxidase subunit 2 (*COX3*); 3) *ND6* suggested in PartitionFinder v1.1.1 [36] were also applied to Partitioned BI analysis respectively with the same alignments to strengthen the results.

Bayesian inference (BI) was performed in MrBayes v3.2 [37,38]. Two separate runs were performed with four Markov chains, each run for 10, 000, 000 generations and sampled every 1000 generations, the consensus tree was calculated after omitting the first 25% trees as burn-in. Maximum likelihood was analyzed in PhyML v3.0 [39]. Basic parameters were estimated with ML methods. A substitution model was also set as GTR from jModeltest result; tree topology search was set as BEST (best of NNI and SPR search). Four relative substitution rate categories were run for 1,000 bootstrap replicates todetermine best tree. We also performed RAxML method on the website http://phylobench.vital-it.ch/raxml-bb/ with GTR+GAMMA model [40] to reconstruct ML phylogenetic trees with default parameters, which yielded a consensus tree with PhyML and BI (Fig 2). The same phylogenetic protocol was applied to construct combined nuclear DNA tree as well as combined mito-nuclear DNA tree with the six nuclear genes. To test the contributions of a single mitogene to the consensus tree, the partitioned Bremer support (PBS) value of every mitogene was calculated in TreeRot V.3 [41]. Every mitogene and gene jackknifing (excluding one gene each run) was also analyzed in BI and the remaining data was used to reconstruct tree. Pairwise Robinson-Foulds distances among trees were calculated with R package phytools [42].

The consensus phylogenetic tree was also used in PAML [43] to detect positive selective loci in mitogenomes of *Arborophila* lineage using branch-sites model A test (model = 2, NSsites = 2) with the clade of hill partridge species set as foreground while the others species were in the background. We run the null hypothesis (fix_momega = 1, omega = 1) and alternative hypothesis (fix_omega = 0, omega = 2) respectively and test the likelihood value (lnL) with chi-square. Bayes Empirical Bayes (BEB) analyses were applied to test positive sites for foreground lineages [44].

**Fig 3:**
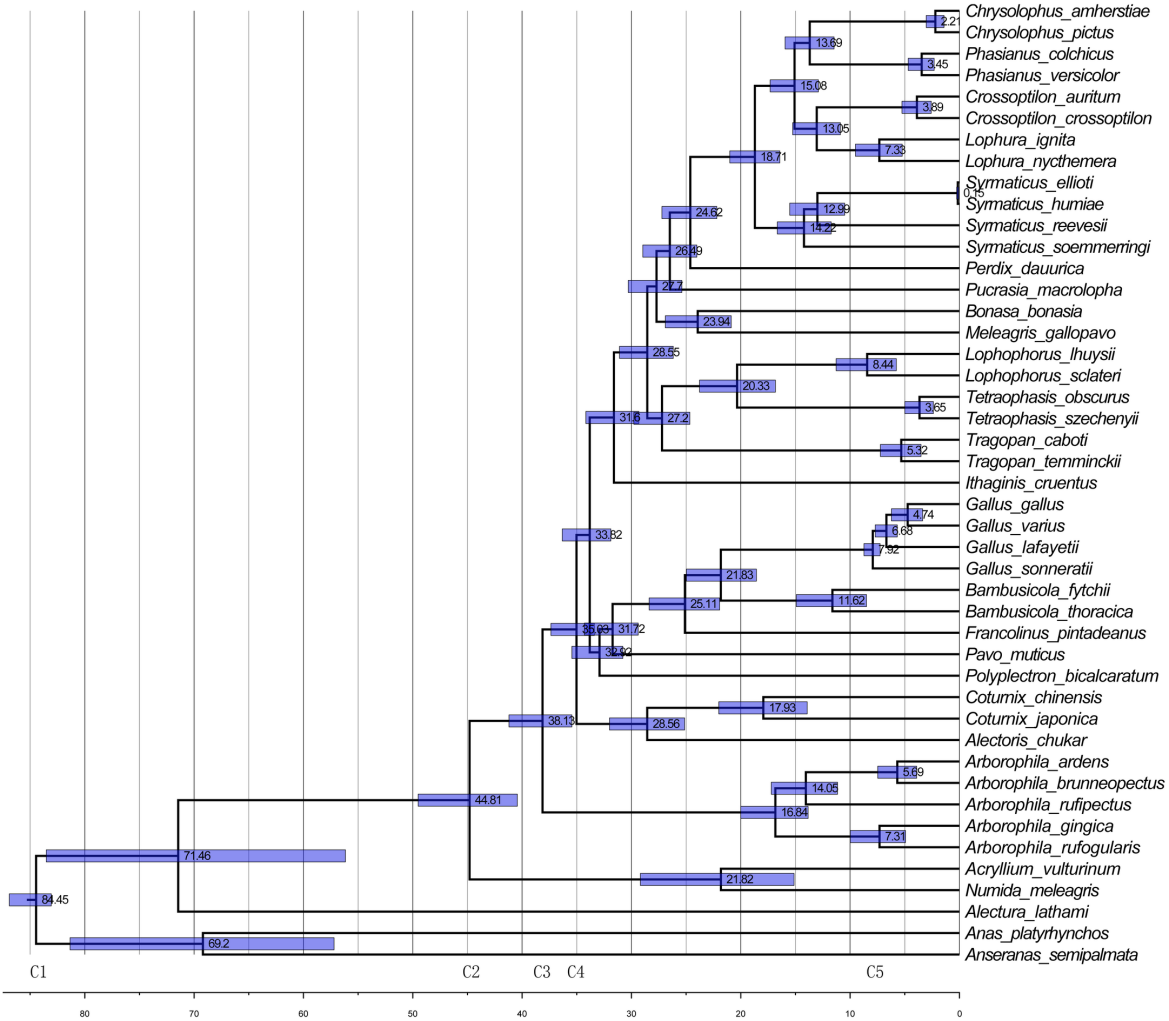
Species tree with estimated divergence time. The divergence times were estimated in BEAST with five fossil calibrations (C1-C5). Mean ages are included beside corresponding nodes. Horizontal gray bars on each node indicate the 95% credible interval of divergence time in millions of years, with the unit of divergence time as Mya.

Fossils are essential as accurate molecular calibrations for constructing modern bird evolutionary timescale [45]. To calculate the divergence time of *Arborophila*, five compatible fossil calibrations (C1~C5 in Fig 3) were chosen to infer Absolute time of each differentiation event: 1) Fowl and ducks diverged at 89.8±7 million years ago (Mya) [46,47]; 2) Numididae*-*Phasianidae split at 52.4±5.1 Mya as the fossil *Gallinuloides wyomingensis* date from 54 Mya [48,49]; 3) Ancestors of *Arborophila* diverged from the other lineages in Phasianidae at around 39 Mya [46,50]; 4) The divergence between the *Coturnix* and *Gallus* was around 35 Mya [46]; 5) *G*. *gallus* and *G*. *sonneratii* diverged around 8 Mya [7]. Divergence times of all 45 birds were estimated in BEAST v2.3.3 [51]. In BEAST, we set uncorrelated lognormal relaxed clock model rate variation among branches. Four of five (C1~C4) compatible fossil calibrations were added to infer absolute time of each differentiation event while C5 was use to verify accuracy and of the inference. 95% confidence intervals included these constraints as calibration priors with a Yule process on the tree. The log-likelihood values and posterior probabilities were checked using TRACER v1.6 [52] to confirm that the chains had become stationary so that the ESS values of our parameters were all high (over 200 or so). The final analysis was run for 10 million generations with trees sampled every 1,000 generations. TreeAnnotator was then used to discard the first 20% trees and to generate the consensus tree. Geographic data of endemic bird areas of the 22 hill partridge species and the other 23 birds were obtained from the BirdLife database [53,54]. Distribution data of the 22 hill partridges were analyzed with R package to produce species distribution maps [55]. Altitude data was also analyzed using R package to produce raster data [56]. The BEAST output consensus tree and endemic bird area geographic data was analyzed in BioGeoBEARS to infer the biogeographic history of phylogenies of the 45 species. We used Dispersal-Extinction-Cladogenesis (DEC) and DEC+J models with AIC method and found no significant difference (p = 0.313) between them.

## Results

### Comparative mitochondrial genomes of *Arborophila*

**Fig 4:**
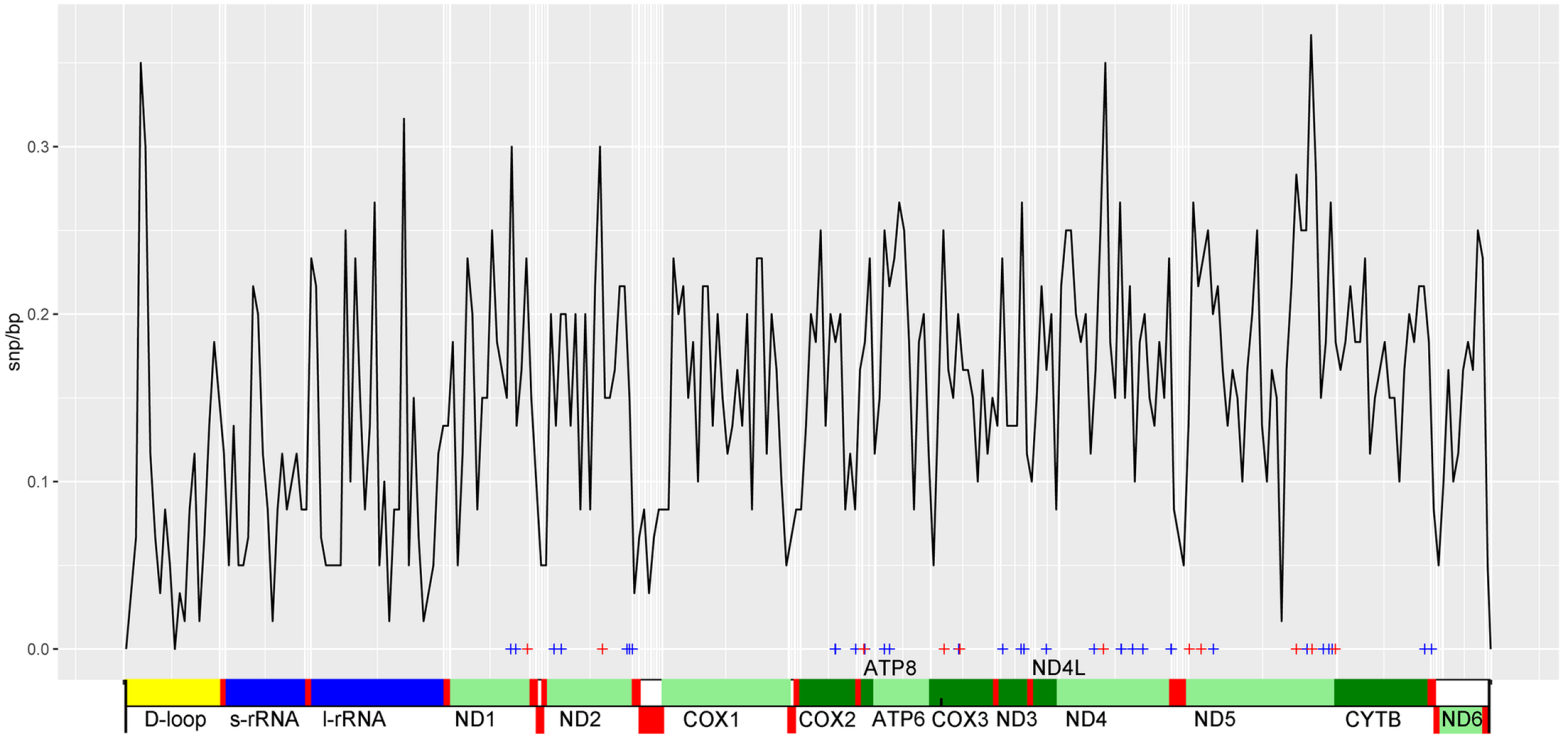
Plot of SNP rates in 60bp-length bins through the whole mitogenome of five hill partridges in this study. Potential positive selective sites detected in PAML are assigned a blue cross, and significant positive selective sites are assigned a red cross.

The newly sequenced mitochondrial genome of *A*. *brunneopectus* is similar to the four previously published *Arborophila* mitochondrial genomes, consisting of 13 protein-coding genes, 22 tRNA genes, two ribosomal RNA genes, and a control region (D-loop) (Genbank accession Number: NC_022684.1) (S2 Table). Most mitochondrial genes are encoded on the H strand, except for the *ND6* gene and eight tRNA genes (tRNA-Gln, tRNA-Ala, tRNA-Asn, tRNA-Cys, tRNA-Tyr, tRNA-Ser (TGA), tRNA-Pro, tRNA-Glu). The base composition of the H strand of the Bar-backed Partridge mt-genome is: 30.40% A; 13.41% G; 24.86% T; 31.3% C, which reflects the typical A-T rich pattern in vertebrate mitochondrial genomes. The length of the *A*. *brunneopectus* mitogenome is 16719bp, which is marginally less than that of the other four hill partridges (16726~16728bp). Insertion/deletion variations occurred mainly in D-loop (loci 215, 219, 227, 298: 1bp per site), 12s rRNA (loci 1937: 6bp del) and 16s rRNA (loci 2834: 7bp del). There are 43 potential positive selective sites (see Fig 4 and S3 Fig) among which 11 sites are significant in mitogene alignments of *Arborophila* compared to that of the other 41 selected species. Notably, rate of variable sites around those potential positive selective loci are significantly higher than the remaining parts of the whole mitogenomes (p < 0.01, Fisher-Pitman Permutation Test). Single-nucleotide polymorphism sites (SNPs) are also intensive in *ND1*, *ND2*, *ND4* and *ND5* other than D-loop and rRNAs. Whereas, the conserved loci are often found in tRNAs and some specific areas of mitogenome components indicating functional importance, such as gene domain of active center of genes. SNPs distributions in *COX1*, *COX2*, *COX3*, *ATP6*, *ATP8* and *CYTB* show more homogeneous than that in the subunits of NADH dehydrogenase.

### The single insertion mutation in *ND3*

**Fig 5:**
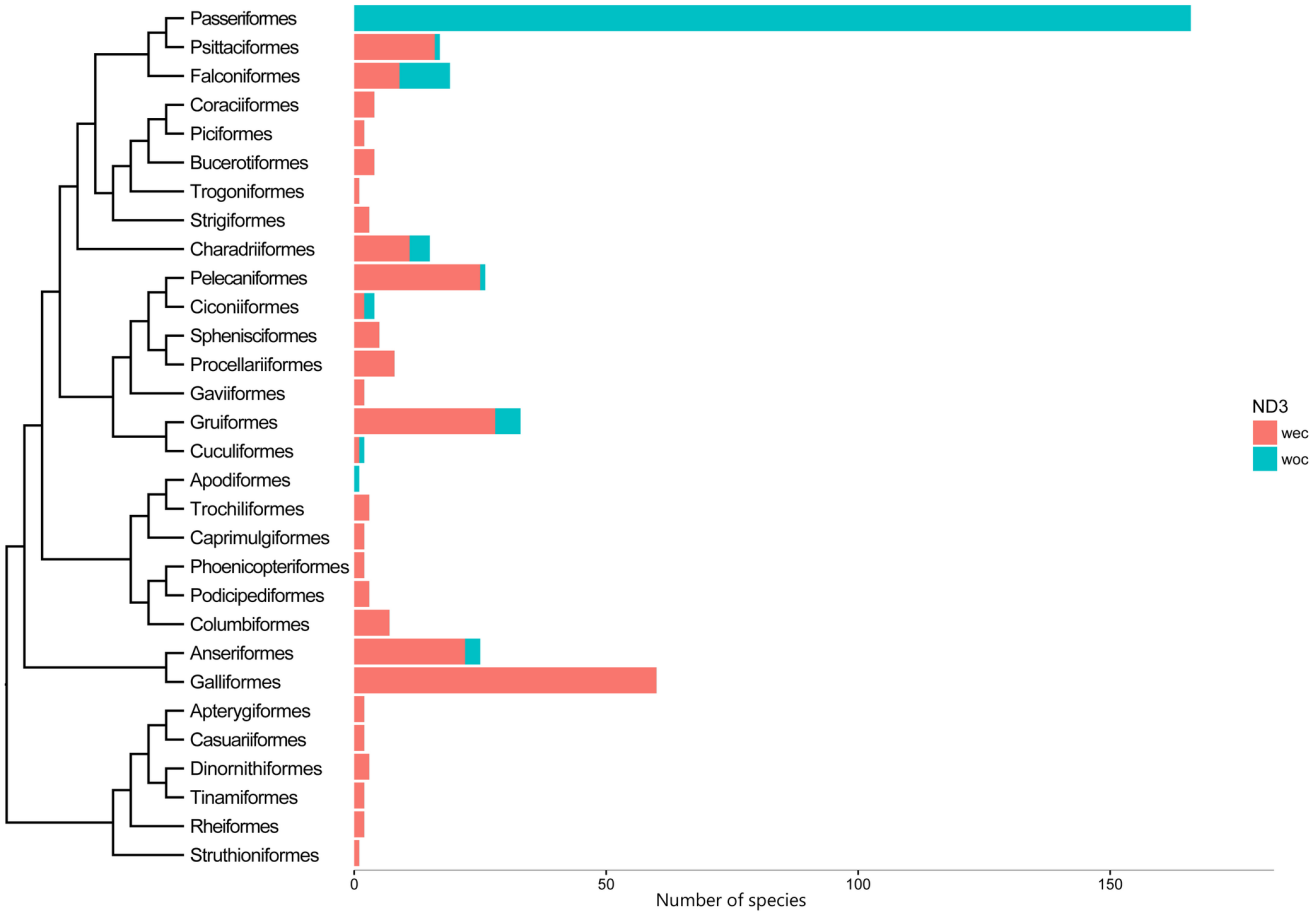
Barplot for counts of *ND3* with extra cytosine (WEC) or without extra cytosine (WOC) in each of the 30 bird orders studied. The data is extracted from 426 species mitogenomes. The phylogenetic relationships of the bird orders are constructed based on three recent studies[24–26].

The insertion of an extra cytosine at position 147 of the *ND3* protein coding sequence that results in premature stopping of translation was confirmed in all the five hill partridge species [57,58]. The extra cytosine insertion information of 426 bird species from 30 orders is depicted in Fig 5. Ten of the 30 orders in our data contain WOC (without extra cytosine) *ND3*. All 166 Passeriformes species and one Apodiformes species have WOC *ND3*, but it is expected that additional information will be found for Apodiformes and thus this is preliminary. Falconiformes, Ciconiiformes and Cuculiformes have nearly equal WOC and WEC (with extra cytosine) *ND3*, while WEC *ND3* is dominant in the other five orders containing both WOC and WEC *ND3*. The near crown taxa shows a tendency to lose the extra cytosine insertion, however, the species without the insertion have not grouped but dispersed in different taxa indicating a complex evolutionary history of birds.

### Phylogeny of *Arborophila* in Phasianidae

Phylogenetic analyses revealed robust tree, with similar topological structures (Fig 2). All Phasianidae species were divided into four deep lineages, as depicted in the consensus tree, among which *Arborophila* was given a basal phylogenetic position, apparently branching earlier than all other Phasianidae genera. The previously suggested taxa (clade I~IX in Fig 2), especially the Old World partridges, were separated and recombined in many situations. Among the five hill partridge species, *A*. *gingica* and *A*. *rufogularis* are a sister-group of a common ancestors shared by of the other three species, *A*. *ardens*, *A*. *brunneopectus* and *A*. *rufipectus*. *Arborophila*. *ardens* and *A*. *brunneopectus* show a closer relationship and diverged later than *A*. *rufipectus*. Such relationships of these five species are defined as type I and supported by two single gene trees (*ND5* and *ND6*). Eight genes (*ATP6*, *COX1*, *COX2*, *CYTB*, *ND2*, *ND3*, *ND4L* and *ND4*) support alternative relationships defined as type II that place *A*. *rufipectus* closer to *A*. *gingica* and *A*. *rufogularis* rather than *A*. *ardens* and *A*. *brunneopectus*. In all single gene trees, *ND5* gene tree has the least distance (RF = 8) relative to the consensus tree. Meanwhile, the tree inferred from combined data exclude *ND5* as it has the biggest distance (RF = 6) compared to the consensus tree (Table 2). The PBS analyses indicated that *ND5* (PBS = 686.83) contributed most to our consensus tree followed by *ND4* (PBS = 554.75) and *ND2* (PBS = 535.58), while *ATP8* (PBS = 21.91) made the least contribution,

**Table 2:** Phylogenetic analyses using jackknifing of single mitogene.

mitogene	PBS	single gene mode			gene jackknifing mode		
		model	topology	RF distance	model	topology	RF distance
ND1	451.67	GTR+I+G	-	36	TVM+I+G	type I	2
ND2	535.58	GTR+I+G	type II	20	GTR+I+G	type I	0
COX1	351.75	GTR+I+G	type II	14	TVM+I+G	type I	0
COX2	160.41	TVM+I+G	type II	28	TVM+I+G	type I	0
ATP8	21.91	GTR+I+G	-	42	TVM+I+G	type I	0
ATP6	290	GTR+I+G	type II	26	TVM+I+G	type I	0
COX3	207	TVM+I+G	-	32	TVM+I+G	type I	0
ND3	51.42	TVM+I+G	type II	28	TVM+I+G	type I	0
ND4L	176	TIM+I+G	type II	36	TVM+I+G	type I	0
ND4	554.75	GTR+I+G	type II	18	GTR+I+G	type I	4
ND5	686.83	TVM+I+G	type I	8	TVM+I+G	type II	6
CYTB	466.16	GTR+I+G	type II	36	TVM+I+G	type I	2
ND6	199.5	K81uf+I+G	type I	28	GTR+I+G	type I	0

PBS: partitioned Bremer support value to the consensus tree; topology refer to clade of *Arborophila*, type I: (((*A*. *arden*, *A*. *brunneopectus*)*A*. *rufipectus*)(*A*.*gingica*, *A*.*rufogularis*)); type II: (((*A*.*gingica*, *A*.*rufogularis*)*A*. *rufipectus*)(*A*. *arden*, *A*. *brunneopectus*)); ‘-‘ means the inferred tree is not typical type I and type II; RF distance: Robinson-Foulds distance of gene tree to the consensus tree.

### Divergence and dispersal

The divergence time calculated from fossil calibrations (C1~C5 in Fig 3) suggests that common ancestors of Phasianidae, *Numida* and *Acryllium* diverged at around 45 Mya. *Arborophila* diverged from Phasianidae 7 Mya later, in Asia, which is consistent with Crow’s and He’s inferences [7,50]. After that time, ancestors of *A*. *rufogularis* and *A*. *gingica* diverged from *Arborophila* at around 16.84 Mya. *A*. *rufipectus* split from ancestors of *A*. *brunneopectus* and *A*. *ardens* at round 14.05 Mya, and these two species diverged at about 5.69 Mya, While *A*. *rufogularis* and *A*. *gingica* diverged at 7.31 Mya. All hill partridge species divergence events occurred during the Miocene when global temperatures underwent gradual cooling.

We collected GIS spatial data of the 22 South-east Asian hill partridge species from BirdLife databases [54]. The distribution areas of the five studied hill partridge species ranged across a wide area with little overlap and often geographically distinct and isolated populations (Fig 1). Only *A*. *brunneopectus* and *A*. *rufogularis* shared overlapping geographical distributions. *Arborophila rufipectus* and *A*. *ardens* represented the north and south, respectively, extents of the five species’ distributions, while *A*. *rufogularis* and *A*. *gingica* represented the west and east (respectively). The geographic distributions of the other 17 south-east Asian hill partridge species were usually across mountainous terrain in Indochina and Maritime Southeastern Asia, where human activities and disturbance can be uncommon. These 17 species only overlapped with the distributions of *A*. *rufogularis* and *A*. *brunneopectus* while are quite distant from the other three studied species(Fig 1). In addition to our phylogenetic analysis of the 45 species, the geographic distribution data allowed a reconstruction of historical/phylogenetic biogeography (S2 Fig) of these species. Historical/phylogenetic biogeography suggests that *Arborophila* split with other Phasianidae species within Asia and, similarly, that the intra-genus divergence likely occurred in Asia.

## Discussion

### SNPs bias positive selective sites within *Arborophila*

It was shown that all positive selection sites experienced nonsynonymous substitution within the five hill partridge species (S3 Fig), whereas, unique AAs were produced from mutations. For example, the mutation ND2: 35V>M (GTA>ATG) occurred in four of five species’ mitogenomes, except *A*. *rufipectus*. It placed Methionine at the site where no Methionine had ever presented. Factors such as high-altitude adaptability [59] may be used to explain the selective but require systematically investigation on ecological data such as the altitude distribution of the Galliformes species. All the positive selection sites might potentially affect the function of NADH dehydrogenase. However, whether enzyme activity of NADH dehydrogenase is different between the studied species or lineages of other hill partridge species and other pheasants requires further verification.

Mitochondrial genes of the studied hill partridge species accumulate SNPs around loci under positive selection relative to other lineages observed. Significantly positive selected sites in *Arborophila* mitogenes are surrounded by a higher level of SNPs rate (proportion of SNP sites in a 60bp-length bin). For example, the SNPs abundant *ND5* contain the most potential positive selective sites (S3 Fig). According to population genetics theory, hitchhiking effort or selective sweep can lead to less genetic diversity around positive selective sites in a population during their fixation. However, whether this effect also applies to individuals from different species living in diverse habitats is uncertain. Every selective site within hill partridge species is composed of different AAs suggests that they might have evolved in different directions. The uneven SNPs distribution in the hill partridge species mitogenomes reflects that selective pressure has not acted equally on the whole mitogenome. The interweaved conserved areas in the mitogenomes could be a specific character for specific lineages, which provides clues of functional domains of a mitogene or conserved sequence boxes in the domain II in D-loop [7]. Additionally, the relative conserved regions of genes may also provide robust and stable markers in the construction of phylogenetic trees, as is noted that the *COXI* or *CYTB* usually yield a consensus tree agreeing with combined data [8]. *ND5* presents a high level of variable sites even compared to D-loop, which indicates that *ND5* may be an alternative in resolving phylogenetic relationships within lower ranks of taxon and may be used intraspecific analysis [12].

### Elimination of cytosine insertion in bird taxa

Clearly, the extra cytosine insertion or +1 frameshift in *ND3* is not a novel phenomenon in *Arborophila* as it is common across bird taxa and absent in others. For example, all species near the root of Palaeognathae have the insertion cytosine, while the younger and highly diverse Passeriformes does not (Fig 5). Nearly all Galloanserae species contain the extra cytosine, except for three Anseriformes species. The exact number/identity of species with and without the extra cytosine requires confirmation, as the uncommon insertion in a mito-gene might be overlooked or regarded as an error in sequencing.

Since many tortoise have the +1 frameshift mutation [58,60], we believe the 147^th^ cytosine insertion is a phenotype inherited from ancestors of all birds. We analyzed the *ND3* insertion in Falconiformes mitogenomes to verify this ancestral heritage of cytosine insertion. Falconiformes is split with 10 WEC *ND3* and 10 WOC *ND3*. The WEC taxa include *Spizaetus*, *Nisaetus*, *Aquila*, *Buteo*, *Accipiter*, *Aegypius* and *Spilornis* and are placed near root while WOC taxainclude *Pandion*, *Sagittarius*, *Falco* and *Micrastur* are near the crown of phylogenetic tree (reconstructed in the same way described in our method). It is hypothesized that certain mitochondrial translation systems have the ability to tolerate frameshift insertions using programmed translational frame shifting [58], but why species in some younger taxa attempt to eliminate the insertion is unclear. One possible interpretation is that mitochondria are material and energetically costly (to sustain normal operations) and mitochondria decrease or offset the influence of frameshift insertion. While species without the deleterious mutation save energy and material and thus is advantageous for these species. It is still unclear why these WOC species dispersed among (relatively) unrelated bird taxa. The loss of an extra cytosine insertion is presumably independent because introgressive hybridization is rare in species from different orders.

### Recent research into phylogenetic discrepancies

The results of our study mirror those of similar recent phylogenetic studies. The clades of the consensus tree formed confounding relationships for pheasants and partridges involving the families: Tetraonidae and Meleagrididae [10,11,28,61,62] (Fig 2). Similarly, our research found that lineages of pheasants and partridges are not monophyletic in as accordance with recent studies [8,62–64]. However recent studies have not been conclusive and the lineages of some species are ambiguous or controversial, such as the blood pheasant (*Ithaginis cruentus*) and peafowl inferred from diverse DNA markers [3,10,11,27,65–67]. Blood pheasant (*Ithaginis cruentus*) was placed at root of taxa composed of gallopheasants, Perdix, tragopans, Tetraoninae and Meleagrididae (BPP = 1; BSP = 0.98) in our consensus tree and some recent studies [10,56–58], but was placed as sister group of Tragopans or at root of family Phasianidae in some other studies [10,59, 62]. Peafowls was commonly placed at root of junglefowls and some Old World partridges lineages as presented in our consensus tree [10,11,56,60,63,64], while some nuclear or combined phylogenetic trees rooted these species within tragopans or even gallopheasants[3,57,58].

Despite this study’s results, the location of hill partridge species’ root at the base of Phasianidae is still controversial. Some nuclear DNA markers, such as CR1 or nuclear ovomucoid intron G place this lineage at a near crown status [9,63]. However, few studies have studied phylogenetic relationships within *Arborophila* [3,12,28,68] akin to this study. Zheng et al. [4] constructed the relationships of 10 hill partridge species in China based on morphology and biogeographic distribution. Chen et al. [68] reconstructed phylogenetic tree of 10 *Arborophila* species with three mito-fragments (CYTB, ND2, COI) and 3 nuclear introns (OVOG, G3PDH, ALDOB). Wang et al. [28] developed trees for seven *Arborophila* species based on two mito-genes and six nuclear intron sequence. The relationships of the five hill-partridge species in our study are consistent with Zheng et al. [4] but are in conflict with Chen et al. [68] and Wang et al. [24] regarding the lineage of *A*. *rufipectus* in *Arborophila*. All four studies agreed regarding the closer relationship between *A*. *ardens* and *A*. *brunneopectus*, which was in accordance with their closer spatial distribution. However, the closely (phylogenetically) related *A*. *gingica* and *A*. *rufogularis* are geographically distant. Interestingly, the geographical distribution of *A*. *rufogularis* overlaps with *A*. *brunneopectus* but they are more phylogenetically remote than to other hill partridge species (Fig 1). The chief inconsistency regarding the relationships of hill partridge species is whether *A*. *rufipectus* shares a closer relationship with *A*. *gingica* and *A*. *rufogularis* or with *A*. *ardens* and *A*. *brunneopectus*. As *A*. *rufipectus* is geographically isolated and far from all other hill partridge species, its phylogeny may be key to discovering the evolutional history of *Arborophila*.

The disparity between Galliformes phylogenetic trees and species-level divergence, and between mitochondrial and ambiguous results may be a result of protein-coding sequence convergence and high levels of incomplete lineage sorting [69] that occurred during their rapid radiation [24]. The disagreements of our consensus tree and previous studies may be due to two reasons. Firstly, evolutionary rates and modes of inheritance are different between nuclear DNA and mitoDNA. With faster evolutionary rates, mitogenes are advantageous for studying relationships of crown groups, while nuclear genes offer abundant information in resolving relationships of deeper clades of a tree. Phylogenetic relationships inferred from nuclear DNA might be bias for taxa in which introgressive hybridization is common [70], while such interference decreases in mitogenomes if it is restricted to male introgression [71]. Secondly, different mitogenes can be discrepant in resolving relationships of species, for evolutionary rate varied through the whole mitogenome. DNA fragments from different sources can have different evolutionary histories in one species.

To further understand which mitogene is responsible for different topologies in *Arborophila* taxa, we carefully reconstructed the phylogenetic trees with combined datasets as well as a single mitogene. The results suggested that *ND5* strongly affected the status of *A*. *rufipectus* within *Arborophila* from combined datasets. *ND5* was demonstrated under positive selection including five of the 11 significant positive selective sites and had faster evolutionary rate. As the longest component in 13-mitogenes combined data, *ND5* contributed most to the consensus tree with the highest PBS value (Table 2). Nevertheless, *ND5*-excluded combined data produced the tree of biggest RF distance relative to consensus tree, while tree inferred from only the *ND5* gene was closest to the consensus tree suggesting its dominance in influencing the topology of the consensus tree. Interestingly, the influence of *ND5* of the consensus tree was restricted to the taxa of *Arborophila*, while it changed the topology of the five hill partridge species from type I to type II. In an additional test, the *ND5* even reversed the phylogenetic tree constructed from combined data of six nuclear DNA fragments, which had strongly supported the type I topology. When adding *ND5* to the combined data, the topology of the five hill partridge species turned from type I to type II (S4 Fig). However, when adding 13 mitogenes to the six nuclear combined data, the mitogenes-nuclear data supported the type I topology, but with rather low Bayesian posterior probabilities (0.56) at node (*A*. *rufipectus* (*A*.*gingica*, *A*.*rufogularis*)). Chen et al. [68] and Wang et al.[28] had not used the *ND5* gene in their phylogenetic analyses. Li et al. [72] reconstructed the phylogenetic tree without the *A*. *brunneopectus*, and they used the whole mitogenomes rather than 13 concatenated mitogenes, and with such treatment the much longer sequences may reduce or offset the influence of *ND5*. The whole mitogenome caused discordance and low support of deep nodes of the consensus tree so was discarded in our phylogenetic analyses.

The dominant influence of *ND5* on *Arborophila* clade topology suggests that the other 12 mitogenes or the six nuclear genes can offer limited phylogenetic information in *Arborophila* taxa. It is, however, still debated whether type I or type II is closer to the true evolutionary lineage of these five hill partridge species. The results of phylogenetic studies may also be affected by different length of gene segment lengths and lack of intermediate species. In this regard, we thought mitogenomes, especially when combined with nuclear DNA, were efficient molecular markers for the study of phylogenetic relationship among Phasianidae species [73–75]. However, cautiou is advised when choosing appropriate nuclear markers, as hybridization is common in bird taxa [73]. In many cases, the phylogenetic relationships of birds become confused when adding nuclear genes [9,11,63]. Compared to single mitochondrial gene sequence, the combined multi-gene sequence of mitogenomes can provide abundant information of the evolutionary relationships of many taxa within Phasianidae [8,76].

### Climate shapes distributions of hill partridges

The divergence time of these five hill partridges were generally earlier in our results than those of Chen’s [68] study. The only exception was that Chen et al. [68] proposed 48.8 Mya as the divergence point for ancestors of *Arborophila* when they split from other Phasianidae species, which was much earlier than our 38.13 Mya. Chen et al. [68] indicated that ancestors of *A*. *ardens* and *A*. *crudigularis* (originating from Indochina) arrived on their islands after the sea level retreat during the last glacial period started at 2.58 Mya [68,77], evolving into new species at approximately 2.2 Mya. However, it is still unclear when *A*. *ardens* diverged from *A*. *brunneopectus* and the natal location of their common ancestor.

The current geographic distribution of species can suggest their evolutionary history [78]. Crowe et al. [50] suggested that there was an initial dispersal by the common ancestor of *Xenoperdix* and *Arborophila* from Africa to Asia. Ning et al. [67] came to a similar conclusion using combined mitogenes and nuclear DNA data. These studies are consistent with the results of our historical/phylogenetic biogeography calculated in BioGeoBEARS (S2 Fig). We further demonstrated that ancestors of *Arborophila* split with other Phasianidae species in Asia and further divided into the diverse hill partridge species. We notice that all four divergnece events happened in the Miocene (23.03 to 5.33 Mya) where there was a moderately warm climate but with two major temperature decreases at 14 Mya and 8 Mya respectively [79–81]. These two global cooling events roughly coincide with the divergence events of *A*. *rufipectus* and *A*. *ardens/A*. *brunneopectus*, and *A*. *gingica* and *A*. *rufogularis* (Fig 3). We propose that climate cooling forced hill partridges species from higher altitudes to lower and migrated to the margins of, and away from, the Yungui and Tibetan Plateaus. Migration to warmer climates during such extreme cooling events may explains why the five studied hill partridge species are isolated and distant from the Tibetan and Yungui Plateaus, leaving a geographic zone devoid of hill partridge species (Fig 1). Since extreme events have caused speciation of hill partridge species, the current habitat fragmentation and geographic isolation may prompt further speciation.

As root genus of Phasianidae, the origin of *Arborophila* is important in determining the inchoate evolutionary history of this family. Similarly, the phylogeny of *Arborophila* can increase the understanding of the geographical distribution and migration of these short-ranging birds who are poor fliers with low aerial maneuverability. However, the status of *A*. *rufipectus* within *Arborophila* is still unclear and it recommended that further analysis of molecular data of *Arborophila* is required.

## Supporting information

S1 Fig Analyses of substitution saturation at each codon position. Maximum likelihood saturation plots were compared between the codon positions of the complete nucleotide dataset. The Y = X line marks the theoretical limit where the number of observed substitutions equals the number of inferred substitutions. The slope of the linear regression indicates the amount of substitution saturation; the smaller the slope, the greater the number of inferred multiple substitutions. The blue ‘x’ and green hollow triangle refer to transition rate and transversion rate of 1^st^ positions of codons respectively; the light blue solid triangle and red hollow triangle refer to transition rate and transversion rate of 2^nd^ positions of codons respectively; the pink solid triangle and yellow hollow square refer to transition rate and transversion rate of 3^rd^ positions of codons respectively; the navy blue solid square and green hollow diamond refer to transition rate and transversion rate of all DNA positions.
(TIF)

S2 Fig Ancestral area reconstruction for Phasianidae and outgroups used in this study. Pie charts in the nodes indicates the probable ancestral areas calculated in BioGeoBEARS using DEC model. Squares at end of each branch are areas the corresponding species inhabited. As: Asia, Af: Africa, Am: America, Eu: Europe, Au: Australia.
(TIF)

S3 Fig Collection of positive selection sites through the mitogenomes. The clade of hill partridges set as foreground and the other 40 birds background in selection pressure analysis. Significantly positive selection sites are signed with red asterisk at the top of the column. Amino acids of large differences in same column are filled with dark yellow.
(TIF)

S4 Fig Molecular phylogenetic tree based on 13 mitochondrial protein-coding genes and six nuclear introns combined dataset using Bayesian inference (BI). The tree contains 35 of 45 species in the 13 combined mitogenes tree. Ignoring absent species in tree from the combined mito-nuclear genes, their topologies are the same except the status of *A*. *rufipectus*. The very low Bayesian posterior probability at that node may result from the discrepancies that mitogenes and nuclear introns yield different topologies at these branches.
(TIF)

S1 Table Amplification and sequencing primers for complete mitochondrial DNA of *Arborophila brunneopectus*. * marks the primers used in LA-PCR, while the remaining primers are used in normal PCR.
(DOCX)

S2 Table Mitochondrial DNA structure of *Arborophila brunneopectus*. ▲mtDNA components, genes of tRNA are signed as their correspond one-letter abbreviation, genes located in L-strand are sign “-”behind their name *Numbers correspond to the nucleotides separating adjacent genes. Negative numbers indicate overlapping nucleotides.
(DOCX)

S3 Table Mitochondrial *ND3* genes information of 426 birds. The states of *ND3* are signed with WOC: Without extra Cytosine and WEC: With extra Cytosine.
(XLSX)
