# Supplementary figures and images for: A novel mitochondrial genome of Arborophila and new insight into Arborophila evolutionary history
Source: PLoS One. 2017 Jul 25;12(7):e0181649. doi: 10.1371/journal.pone.0181649 (PMC5526529; doi:10.1371/journal.pone.0181649)

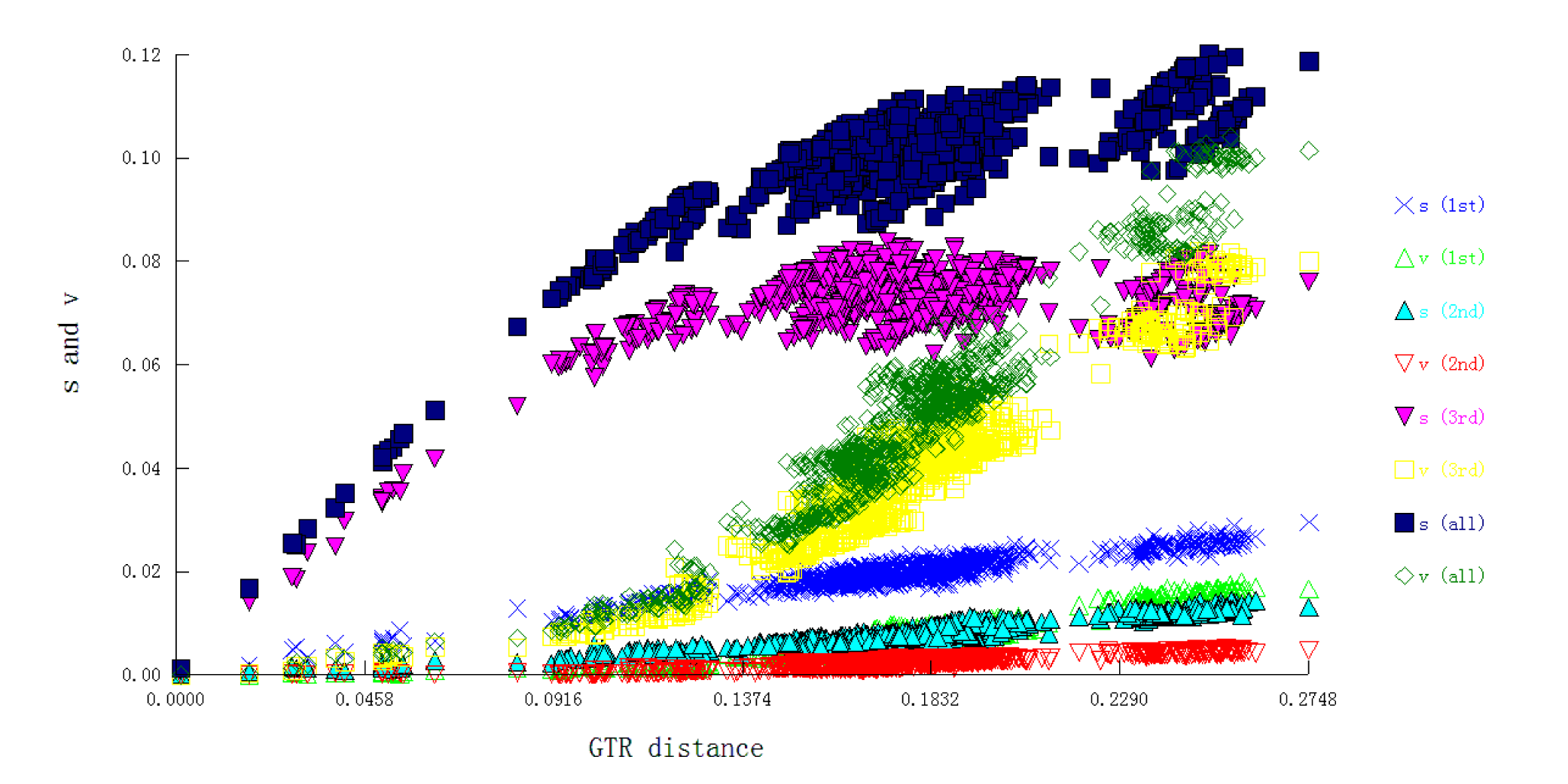

Supplement: S1 Fig — Maximum likelihood saturation plots were compared between the codon positions of the complete nucleotide dataset. The Y = X line marks the theoretical limit where the number of observed substitutions equals the number of inferred substitutions. The slope of the linear regression indicates the amount of substitution saturation; the smaller the slope, the greater the number of inferred multiple substitutions. The blue ‘x’ and green hollow triangle refer to transition rate and transversion rate of 1st positions of codons respectively; the light blue solid triangle and red hollow triangle refer to transition rate and transversion rate of 2nd positions of codons respectively; the pink solid triangle and yellow hollow square refer to transition rate and transversion rate of 3rd positions of codons respectively; the navy blue solid square and green hollow diamond refer to transition rate and transversion rate of all DNA positions. (TIF) [file pone.0181649.s001.tif]

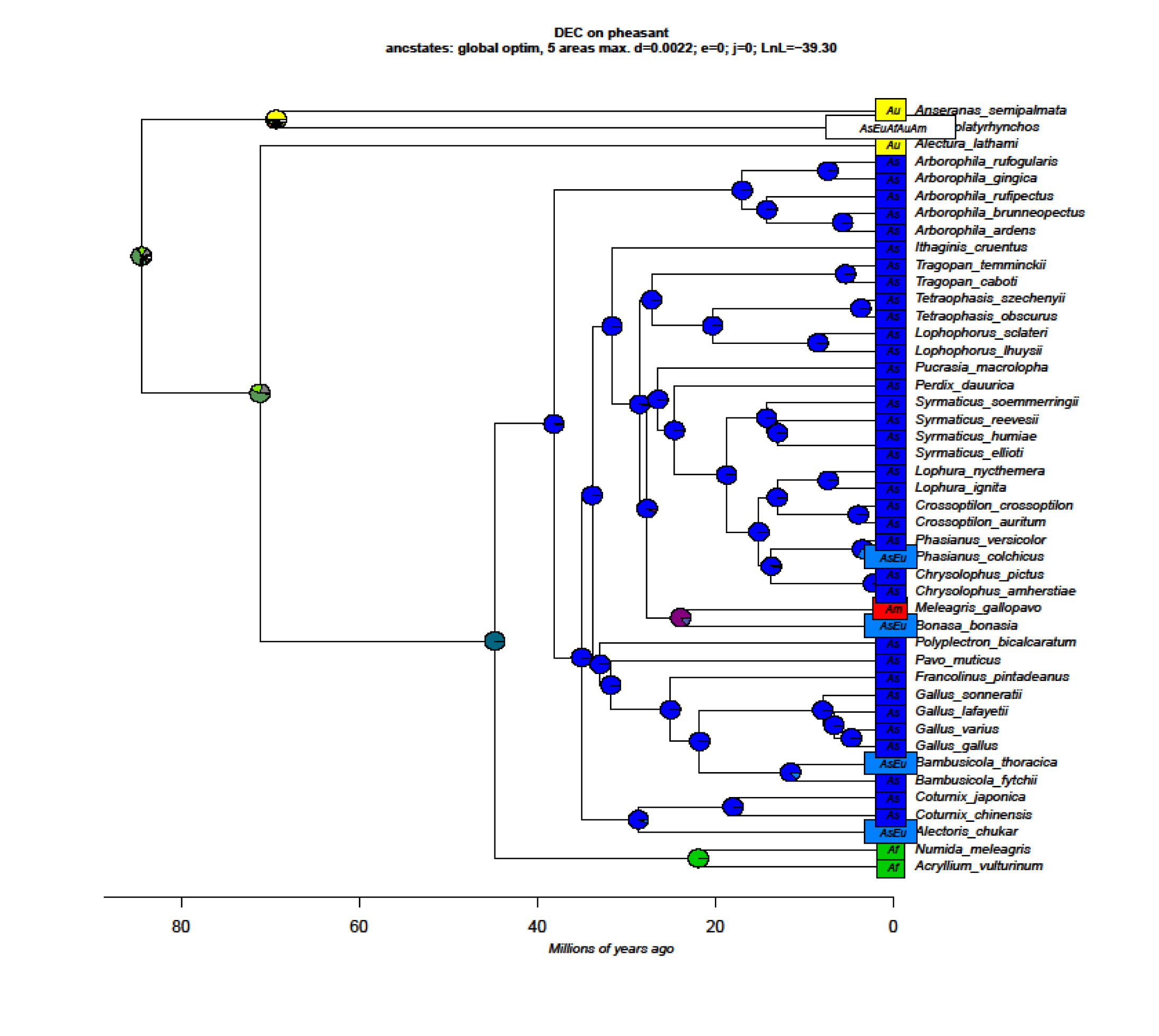

Supplement: S2 Fig — Pie charts in the nodes indicates the probable ancestral areas calculated in BioGeoBEARS using DEC model. Squares at end of each branch are areas the corresponding species inhabited. As: Asia, Af: Africa, Am: America, Eu: Europe, Au: Australia. (TIF) [file pone.0181649.s002.tif]

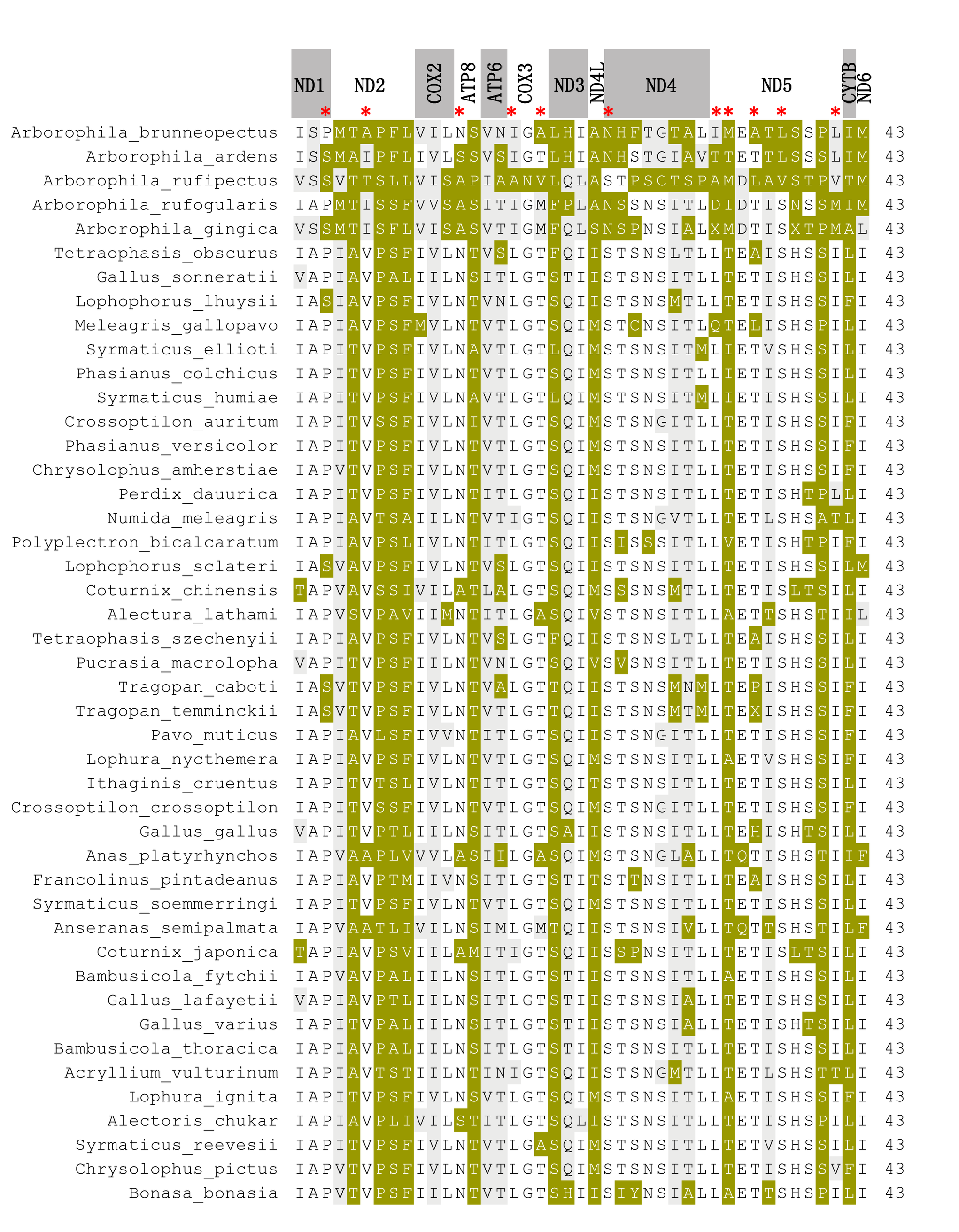

Supplement: S3 Fig — The clade of hill partridges set as foreground and the other 40 birds background in selection pressure analysis. Significantly positive selection sites are signed with red asterisk at the top of the column. Amino acids of large differences in same column are filled with dark yellow. (TIF) [file pone.0181649.s003.tif]

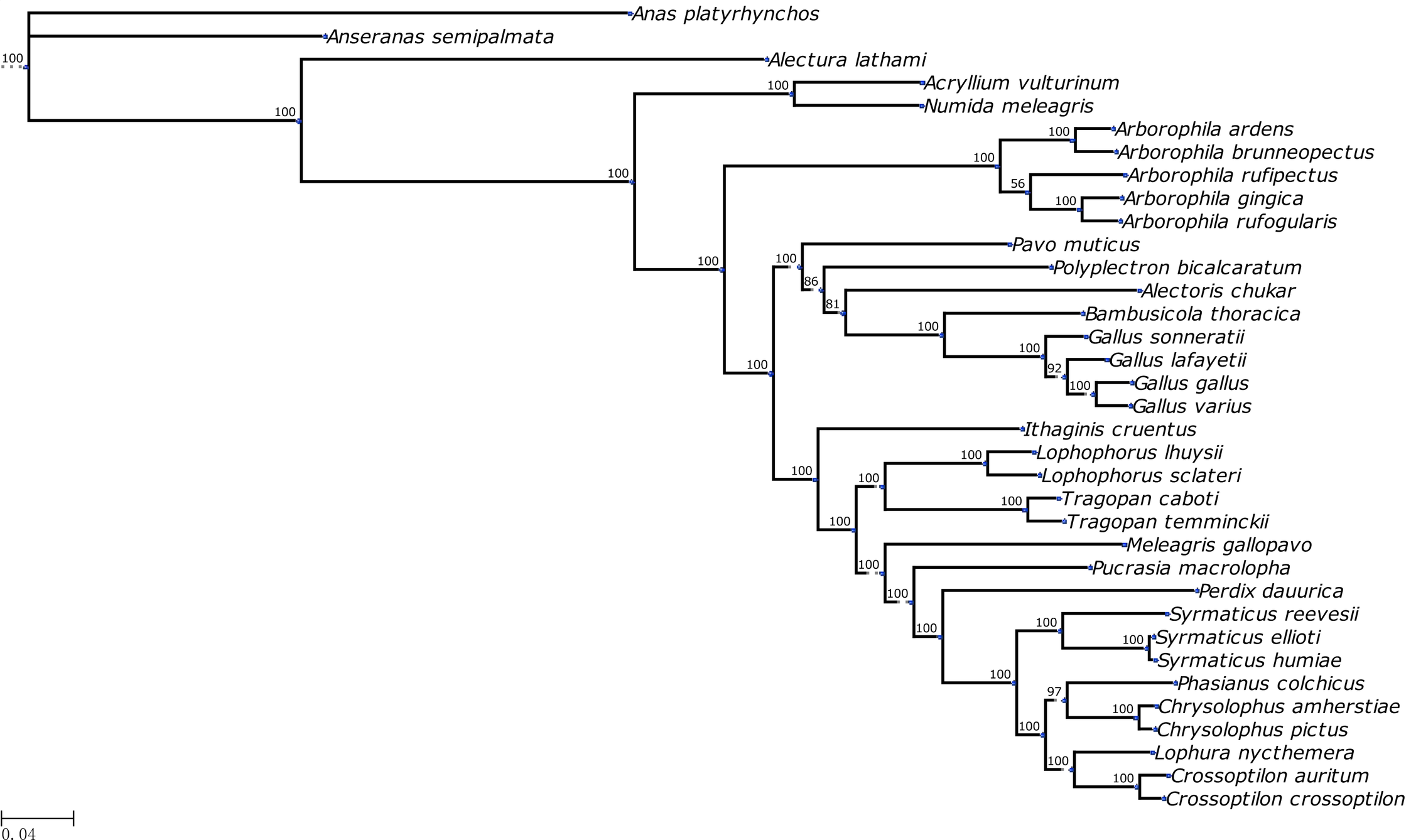

Supplement: S4 Fig — The tree contains 35 of 45 species in the 13 combined mitogenes tree. Ignoring absent species in tree from the combined mito-nuclear genes, their topologies are the same except the status of A. rufipectus. The very low Bayesian posterior probability at that node may result from the discrepancies that mitogenes and nuclear introns yield different topologies at these branches. (TIF) [file pone.0181649.s004.tif]
