# Supplementary material for: A novel mitochondrial genome of Arborophila and new insight into Arborophila evolutionary history
Source: PLoS One. 2017 Jul 25;12(7):e0181649. doi: 10.1371/journal.pone.0181649 (PMC5526529; doi:10.1371/journal.pone.0181649)
Supplement: S1 Table — * marks the primers used in LA-PCR, while the remaining primers are used in normal PCR. (DOCX) [file pone.0181649.s005.docx]

**S1 Table.** **Amplification and Sequencing Primers for complete mitochondrial DNA of Arborophila brunneopectus.**

| primer name | primer sequences | source |
| --- | --- | --- |
| AAR1_F / AAR1_R* | ACCATTATTAACATAAAACCCC / TAGCTTAAAAGGCTAGTGCTG | Sorensen,1999 |
| AAR2_F / AAR2_R* | GTAACAAGGTAAGTGTACCGGAAGG / GCTAGGGAGAGGATTTGAACCTC | Sorensen,1999 |
| AAR3_F / AAR3_R* | TTCGAAGCAACCCTAATCCCAAC / AGGCCAAATTGAGCGGATTTTCC | Sorensen,1999 |
| AAR4_F / AAR4_R* | ACCGCCAACCTTCTTATCCT / GGCTACTGTTGTTTGGTGTGC | He,2009 |
| AAR5_F / AAR5_R* | GGTCGGTTTCTATCTATGTATGC / GTTTCGGTCGGTGAGTAGTATT | He,2009 |
| AAR6_F / AAR6_R* | GACCAGGAGTGTTTTACGGAC / TTAAGGAGAGGCAGGTGGAG | He,2009 |
| AAR7_F / AAR7_R* | CACACCTAATCAATCCAGCG / ATACTGGGACGGAGTTAGAATGT | He,2009 |
| AAR8_F / AAR8_R* | CGTAGGATGAGAGGGAGTAGGA / TGCTGAAGTATGAAGTGGATGC | He,2009 |
| AAR6<1>F / AAR6<1>R | TAGTCCCAACAACAGACCTTCC / CGATTATGGCTACGGCTACTTC | this study |
| AAR6<2>F / AAR6<2>R | CCAGAACACCGATGAATCAACAA / AGGAAGAATGATCCGATGATAACG | this study |
| AAR6<3>F / AAR6<3>R | TTTCCTTTGTTGCCACAGGATT / AATTCGTATAATGCCATATCCTC | this study |
| AAR8<1>F / AAR8<1>R | GGCTGATGACAAGGACGAG / TGGGTGCCATTAGGTTATTT | this study |
| AAR8<2>F / AAR8<2>R | GCCTAATCAAGCCCTATCTA / TTCTAAGGTTCCGATCATGG | this study |
| AAR8<1_2>F / AAR8<1_2>R | AGGCTTACTAATCTCAACCCTC / ATTCTACTAGGGTTTGTCCGAT | this study |
| AAR<5_1>F / AAR<5_1>R | ACCTACCGCCATCCTAAC / TCGGGTGTCTACGTCTATTC | this study |
| AAR<6_3>F / AAR<6_3>R | AGAGCACATTCCAAGGTCA / TACAAAGTGTCAGTATCAGGC | this study |
| AAR<7_2>F / AAR<7_2>R  AARB<3_8>F / AARB<3_8>R  AARB4_F / AARB4_R  AARB5_F / AARB5_R | TGGGAACTACGAGCACAAACG / GGATGAGGGAGTCAAGTGGGA  TCCAAACTTAAACACACGAGA / TCCGATTGTAACTATTATTAGGC  GCTTACGCTATTCTACGCTCTA / GACACCATCTTGGCATCTTC  TGATTCCGCTACGACCAACTA / CCAAAGATTAGGTAAAGAGTGCC | this study  this study  this study  this study |

* marks the primers used in LA-PCR, while the remaining primers are used in normal PCR.
