# Supplementary material for: A novel mitochondrial genome of Arborophila and new insight into Arborophila evolutionary history
Source: PLoS One. 2017 Jul 25;12(7):e0181649. doi: 10.1371/journal.pone.0181649 (PMC5526529; doi:10.1371/journal.pone.0181649)
Supplement: S2 Table — ▲mtDNA components, genes of tRNA are signed as their correspond one-letter abbreviation, genes located in L-strand are sign “-”behind their name *Numbers correspond to the nucleotides separating adjacent genes. Negative numbers indicate overlapping nucleotides. (DOCX) [file pone.0181649.s006.docx]

**S2 Table.** **Mitochondrial DNA structure of *Arborophila brunneopectus*.**

|  | Arborophila brunneopectus | | | |
| --- | --- | --- | --- | --- |
| component sign▲ | start..end | Promoter/ anticode | terminater | intergenic nucleotide |
| D-LOOP | 1..1174 |  |  | 0 |
| F | 1175..1242 | GAA |  | 0 |
| 12S | 1242..2215 |  |  | -1 |
| V | 2216..2289 | TAC |  | 0 |
| 16S | 2291..3912 |  |  | 1 |
| L | 3913..3986 | TAA |  | 0 |
| *ND1* | 3993..4967 | *ATG* | *TAA* | *6* |
| I | 4968..5038 | GAT |  | 0 |
| Q- | 5114..5044 | TTG |  | -1 |
| M | 5114..5182 | CAT |  | 5 |
| *ND2* | 5183..6223 | *ATG* | *TAG* | *0* |
| W | 6222..6297 | TCA |  | -2 |
| A- | 6372..6304 | TGC |  | 6 |
| N- | 6447..6375 | GTT |  | 2 |
| C- | 6516..6450 | GCA |  | 2 |
| Y- | 6585..6516 | GTA |  | -1 |
| *COX1* | 6587..8137 | *GTG* | *AGG* | *1* |
| S- | 8203..8129 | TGA |  | -9 |
| D | 8206..8274 | GTC |  | 2 |
| *COX2* | 8276..8959 | *ATG* | *TAA* | *1* |
| K | 8961..9028 | TTT |  | 1 |
| *ATP8* | 9030..9194 | *ATG* | *TAA* | *1* |
| *ATP6* | 9185..9868 | *ATG* | *TAA* | *-10* |
| *COX3* | 9868..10651 | *ATG* | *T* | *-1* |
| G | 10652..10721 | TCC |  | 0 |
| *ND3* | 10722..11073 | *ATG* | *TAA* | *0* |
| R | 11072..11143 | TCG |  | -2 |
| *ND4L* | 11144..11440 | *ATG* | *TAA* | *0* |
| *ND4* | 11434..12811 | *ATG* | *T* | *-7* |
| H | 12812..12880 | GTG |  | 0 |
| S | 12882..12945 | GCT |  | 1 |
| L' | 12947..13017 | TAG |  | 1 |
| *ND5* | 13018..14832 | *ATA* | *TAA* | *0* |
| CYTB | 14837..15979 | ATG | TAA | 4 |
| T | 15982..16050 | Thr |  | 2 |
| P- | 16122..16053 | TGG |  | 2 |
| *ND6* | 16129..16650 | *ATG* | *TAG* | *6* |
| E- | 16719..16652 | TTC |  | 1 |

▲mtDNA components, genes of tRNA are signed as their correspond one-letter abbreviation, genes located in L-strand are sign “-”behind their name. Numbers of intergenic nucleotide correspond to the nucleotides separating adjacent genes. Negative numbers indicate overlapping nucleotides.
